# Insights into Biological Role of LncRNAs in Epithelial-Mesenchymal Transition

**DOI:** 10.3390/cells8101178

**Published:** 2019-09-30

**Authors:** Jun-Ting Cheng, Lingzhi Wang, Hong Wang, Feng-Ru Tang, Wen-Qi Cai, Gautam Sethi, Hong-Wu Xin, Zhaowu Ma

**Affiliations:** 1 School of Basic Medicine, Health Science Center, Yangtze University, Nanhuan Road, Jingzhou, Hubei 434023, China; chengjunting1001@163.com (J.-T.C.); cwq7537@163.com (W.-Q.C.); hongwu_xin@126.com (H.-W.X.); 2 The First School of Clinical Medicine, Health Science Center, Yangtze University, 1 Nanhuan Road, Jingzhou, Hubei 434023, China; 3 Cancer Science Institute of Singapore, National University of Singapore, Singapore 117599, Singapore; csiwl@nus.edu.sg; 4 Department of Pharmacology, Yong Loo Lin School of Medicine, National University of Singapore, Singapore 117600, Singapore; 5 Singapore Nuclear Research and Safety Initiative, National University of Singapore, Sinagpore 138602, Singapore; snrwh@nus.edu.sg (H.W.); snrtfr@nus.edu.sg (F.-R.T.)

**Keywords:** long non-coding RNA, epithelial-mesenchymal transition (EMT), cancer, signaling pathways

## Abstract

Long non-coding RNAs (lncRNAs) are versatile regulators of gene expression and play crucial roles in diverse biological processes. Epithelial-mesenchymal transition (EMT) is a cellular program that drives plasticity during embryogenesis, wound healing, and malignant progression. Increasing evidence shows that lncRNAs orchestrate multiple cellular processes by modulating EMT in diverse cell types. Dysregulated lncRNAs that can impact epithelial plasticity by affecting different EMT markers and target genes have been identified. However, our understanding of the landscape of lncRNAs important in EMT is far from complete. Here, we summarize recent findings on the mechanisms and roles of lncRNAs in EMT and elaborate on how lncRNAs can modulate EMT by interacting with RNA, DNA, or proteins in epigenetic, transcriptional, and post-transcriptional regulation. This review also highlights significant EMT pathways that may be altered by diverse lncRNAs, thereby suggesting their therapeutic potential.

## 1. Introduction

Long non-coding RNAs (lncRNAs) are a large class of regulatory transcripts longer than 200 nucleotides lacking evident protein coding potential [1,2]. LncRNAs execute a broad repertoire of functions involving multiple biological processes such as imprinting genomic loci, shaping chromosome conformation, and allosterically regulating enzymatic activity. During the latest decade, mounting evidence has revealed expanding roles for lncRNAs in physiological and pathological processes, including cancer cell biology [3,4,5,6]. Numerous studies have shown that lncRNA dysregulation plays key roles in human diseases, including cancer, by modulating the epithelial-mesenchymal transition (EMT). With this growing appreciation for lncRNAs as crucial EMT regulators, the need for lncRNA-based treatments for various diseases is becoming apparent.

EMT is a cellular program wherein cells lose their epithelial features and acquire mesenchymal characteristics, which enables them to migrate more effectively and invade the underlying mesenchyme [7]. However, the entirety accomplishment of the EMT process requires an intricate genetic procedure [8]. EMT can be utilized by both normal and tumor epithelial cells to enable them to separate from neighboring cells and migrate [8]. Transcriptional and epigenetic profiles have been used to identify underlying gene-regulatory networks, transcription factors, and signaling pathways that control diverse EMT states [9]. Accumulating evidence indicates that lncRNAs can be implicated in phase transition and cellular compartmentalization. For instance, NEAT1 serves as an essential architectural component of paraspeckle nuclear bodies [10]. Additionally, certain well-known lncRNAs (such as HOTAIR, MALAT1, and MEG3) have been identified as master regulators of EMT-related transcription factors, including members of the Snail, ZEB, and Twist families [11,12,13,14,15]. Understanding lncRNA regulation of EMT, therefore, may improve diagnosis and therapeutics for a range of diseases.

Recent reviews have focused on the emerging roles of lncRNAs and EMT in cancers and metastasis [16,17,18,19]. The main objective of this review is to provide an overview of the expanding landscape of lncRNAs across all EMT types. We highlight the multifaceted functions of lncRNAs and their underlying molecular mechanisms, with a special focus on cross-talk between signaling pathways.

## 2. LncRNAs as Emerging EMT Regulators

**Figure 1:**
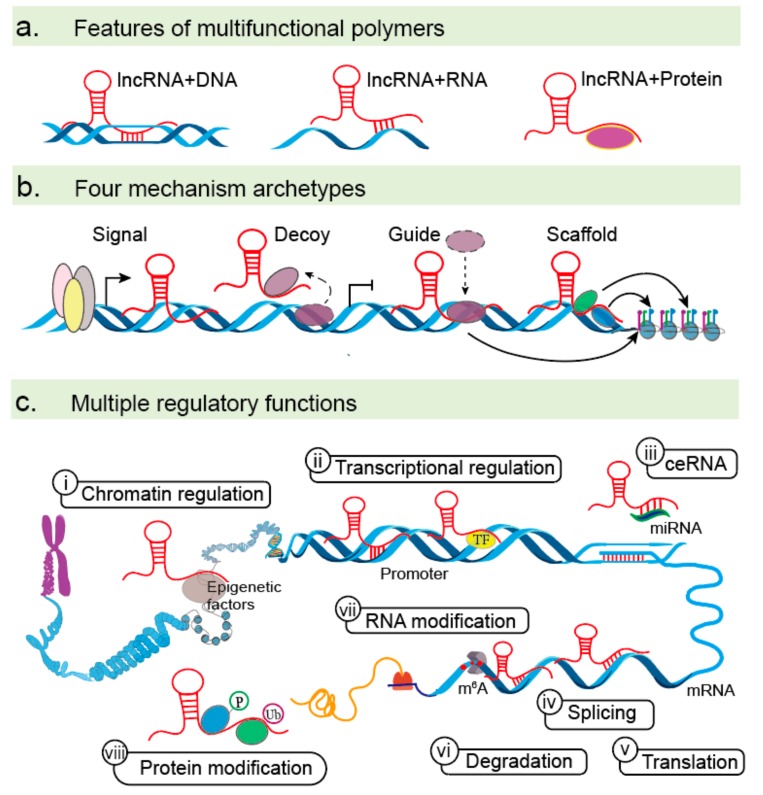
LncRNA functions. (**a**) lncRNAs can bind DNA, RNA, and protein molecules to regulate gene expression at multiple levels via base pairing or secondary structure formation. (**b**) LncRNAs have four primary roles as signals, decoys, guides, and scaffolds. (**c**) Mechanism of action for lncRNAs in the nucleus (**i–ii**) and cytoplasm (**iii–viii**). (**i**) LncRNAs can recruit epigenetic factors to change patterns of chromatin organization, (**ii**) activate or repress the transcription of certain genes by interacting with DNA sequences or TFs, (**iii**) act as ceRNAs by base pairing with miRNA and diminishing its inhibitory effects, and manipulate mRNA function by base pairing to (**iv**) regulate alternative splicing (e.g., MALAT 1), (**v**) affect mRNA translation (e.g., TTN-AS1 and AC132217.4), and (**vi**) mRNA degradation (e.g., CASC11). (**vii–viii**) lncRNAs can modify mRNA and proteins, playing regulatory roles in methylation, phosphorylation, and ubiquitination.

A schematic of the general features, functions, and mechanisms of action of lncRNAs is provided as a background (Figure 1). LncRNAs are a large and heterogeneous class of non-coding RNAs that exert regulatory role by nucleotide base pairing or specific structures generated by RNA folding [1,2,6]. Firstly, lncRNAs play diverse regulatory functions by binding DNA, RNA, and protein molecules [20] (Figure 1a). Their myriad roles in the regulation of gene expression can be classified into four archetypes, including signaling, decoy, guide, and scaffold functions [21,22,23] (Figure 1b). As signaling molecules, lncRNAs can serve as spatiotemporal indicators of gene regulation that reflect the biological effects of transcription factors (TFs) or signaling pathways. As decoys, lncRNAs can sequester TFs and other proteins away from chromatin or into nuclear subdomains. As guides, lncRNAs can recruit RNA binding proteins to target genes, either in cis or in trans. As scaffolds, lncRNAs can recruit various proteins to form complexes with specialized biological functions. Thirdly, lncRNAs play multiple regulatory functions due to their complexity and flexibility [23] (Figure 1c). In the nucleus, lncRNAs can bind epigenetic factors to change chromatin-organizational patterns. LncRNAs also activate or repress transcription of target genes by interacting with DNA sequences or TFs. In the cytoplasm, lncRNAs can act as competing endogenous RNAs (ceRNAs) to compete together with microRNAs (miRNAs) and impair the gene expression of miRNA targets at the transcriptional level. Furthermore, lncRNAs can directly interact with mRNAs to alter their stabilities. LncRNAs can also play vital roles in determining mRNA and protein modifications, such as N6-methyladenosine (m^6^A), protein phosphorylation and ubiquitination [24,25,26,27,28,29]. Together, multifunctions of lncRNAs include chromatin regulation, transcriptional activation and repression, ceRNAs, mRNA regulation, and RNA or protein modifications. With advances in the field of lncRNAs, a new appreciation for the various mechanisms used to control multiple stages of EMT is emerging.

**Figure 2:**
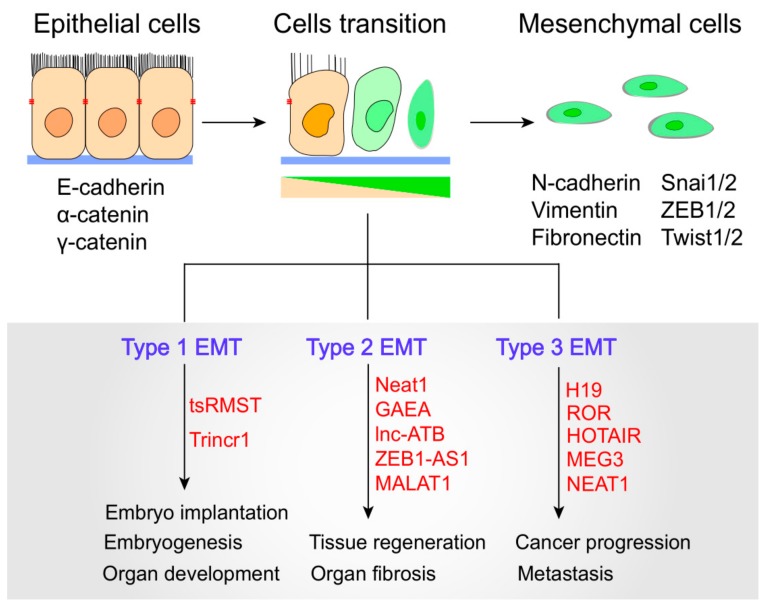
LncRNAs modulate three Epithelial-mesenchymal transition (EMT) subtypes. EMTs involve the functional transition of polarized epithelial cells into mobile and secretory mesenchymal cells. Cells transition indicates progressive loss of epithelial markers and gain of mesenchymal markers. Epithelial and mesenchymal cell markers and related lncRNAs are shown.

Recent studies have revealed that lncRNA dysregulation is associated with EMT in a wide spectrum of physiological and pathological processes. EMTs can be divided into three main biological subtypes. Type-1 EMTs are related to implantation, embryo formation, and organ development and are organized to generate diverse cell types with common mesenchymal phenotypes. Type-2 EMTs are related to wound healing, tissue regeneration, and organ fibrosis. Type-3 EMTs occur in neoplastic cells that have previously undergone genetic and epigenetic changes, especially in terms of genes that favor clonal outgrowth and localized tumor development [7] (Figure 2). Importantly, type-3 EMTs can affect oncogenes and tumor-suppressor genes, thus influencing invasive and therapy resistance property of cancer cells, and thereby generating the life-threatening manifestations of cancer progression [7,30]. To date, lncRNAs have emerged to act as important modulators in a multitude of cellular events ranging from embryogenesis to cell-fate determination. LncRNAs have been shown to modulate all types of EMTs by directly or indirectly interacting with EMT-related molecules. For example, in human embryonic stem cells, the trans-spliced noncoding RNA RMST (tsRMST) downregulates and suppresses Type-1 EMT and embryonic stem cell differentiation by reducing WNT5A expression [31]. Another example is Trincr1 (TRIM71 interacting long noncoding RNA 1), that can promote embryonic stem cells self-renewal and suppress ERK target genes through inhibiting TRIM71 [32]. Recent studies have indicated that lncRNAs can manipulate Type 2 EMT to regulate tissue regeneration and organ fibrosis, such as lncRNA Neat1, MALAT1 and lnc-ATB [33,34,35]. For example, Neat1 can be widely expressed in many tissues and play a key role in muscle cell formation, muscle regeneration and cancer cell proliferation. Neat1 inhibits P21 expression by recruiting Ezh2 to increase the level of H3k27me3 binding at the P21 promoter, thus resulting in the promotion of myoblast proliferation [35]. Moreover, MALAT1 and lnc-ATB can promote EMT during silica-induced pulmonary fibrosis by competitively binding miR-503 and miR-200c, respectively [33,34]. Dysregulated lncRNAs have been found to target diverse type 3 EMT-related genes and signaling pathways to increase epithelial plasticity. Specifically, the lncRNA ROR controls multiple signaling pathways involved in breast, bladder, and nasopharyngeal EMT [36,37,38]. 

We have summarized the roles of various lncRNA that have been confirmed by functional studies in Table 1. A better understanding of lncRNA-dependent EMT regulation may reveal novel targets with therapeutic or prognostic value for various human diseases. 

## 3. The Molecular Mechanisms of lncRNAs in EMTs

### 3.1. LncRNAs Acting on EMTs at the Epigenetic and Transcriptional Levels

#### 3.1.1. Chromatin Modification and Regulation

**Figure 3:**
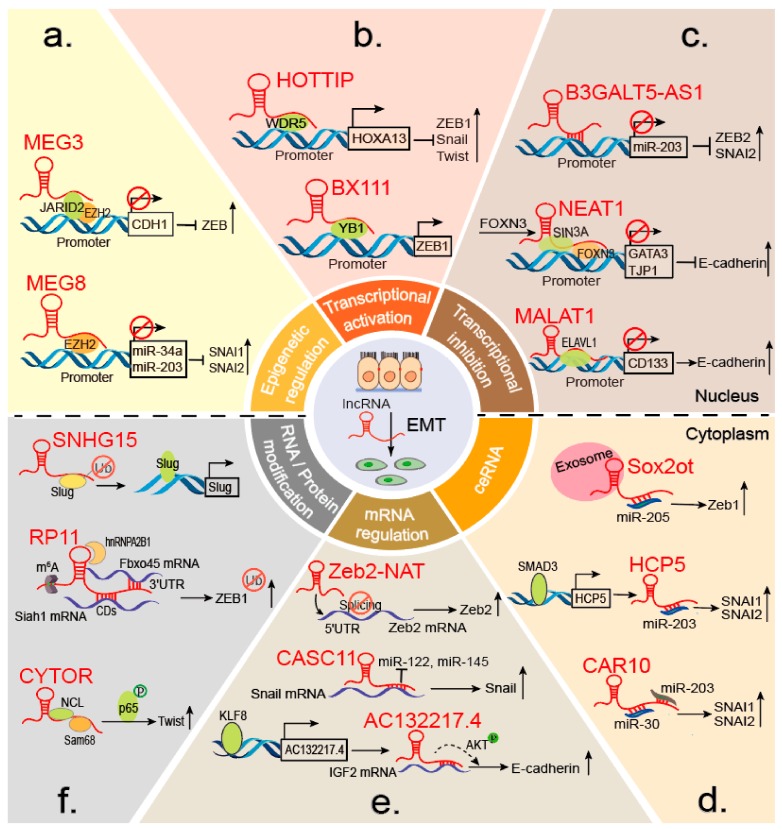
LncRNAs regulate EMT at different levels. (**a**) LncRNAs regulate RNA-protein interactions at the epigenetic level. Both MEG8 and MEG3 suppress the expression of downstream target genes by interacting with EZH2, resulting in EMT marker upregulation. (**b–c**) During transcription, lncRNAs function via RNA-TF or RNA-DNA (e.g., B3GALT5-AS1) interactions. LncRNAs act as guides and molecular scaffolds for TF activation (e.g., HOTTIP and BX111) or target gene repression (e.g., MALAT1 and NEAT1) to regulate EMT-related genes such as ZEB1 and E-cadherin. Furthermore, lncRNA B3GALT5-AS1 directly binds the miRNA-203 promotor to repress miR-203 expression, upregulate SNAI2 and ZEB2, and induce EMT. (**d**) LncRNAs (e.g., CAR10 and HCP5) and exosomal lncRNAs (e.g., Sox2ot) act as ceRNAs by competitively binding miRNAs to increase EMT TF expression. (**e**) LncRNAs affect mRNA splicing (e.g., Zeb2-NAT) and stability (e.g., lnc-ATB and AC132217.4) to modulate EMT. (**f**) LncRNAs regulate protein and mRNA modifications to manipulate EMT. They also act as scaffolds to recruit proteins and impact protein phosphorylation and ubiquitination (e.g., SNHG15 and CYTOR). Additionally, m^6^A methylation can induce lncRNA expression (e.g., RP11) by increasing lncRNA accumulation in nuclei.

Accumulating studies have shown that lncRNAs can interact with chromatin-modifying enzymes to stimulate or silence gene expression [63]. EZH2 is the most common epigenetic factor that influence indirectly EMT markers through interacting with lncRNAs. For example, a well-known lncRNA, HOTAIR, is essential for cancer metastasis, is located in the HOXD locus and displays cell-type and tissue-specific expression [64]. In gastric cancer (GC), HOTAIR epigenetically silences miR-34a by binding the PRC2 (polycomb repressive complex 2) component EZH2 to promote EMT progression [65]. The PRC2 complex is indispensable for epigenetic silencing during normal development and cancer [66,67]. Similarly, in lung and pancreatic cancer, MEG8 binds specifically to EZH2 and suppresses miR-34a and miR-203 gene expression, resulting in SNAI1 and SNAI2 upregulation [15] (Figure 3a). Additionally, in TGF-β-induced EMT of lung cancer, MEG3 interacts with EZH2 and JARID2 at the regulatory regions of CDH1 and miR-200 family genes, resulting in transcriptional repression and ZEB upregulation [39] (Figure 3a). MALAT1 in renal cell carcinoma (RCC) and CASC15 in GC induce EMT by decreasing the level of E-cadherin [13,68]. In contrast, ANCR suppresses EMT in breast cancer (BC) by actively regulating E-cadherin via the WNT (Wingless/Integrated) pathway [41]. Together, these findings indicate that lncRNAs epigenetically orchestrate EMT and broaden our understanding of lncRNA-mediated molecular mechanisms involved in cancer initiation and malignancy progression.

LncRNAs have been studied most extensively in the context of other epigenetic factors, such as CDX2 (Caudal-related homeobox 2), ARID1A (AT-rich interaction domain 1A), and LOXL2 (lysyl oxidase-like 2). LINC00518 binds to the CDX2 gene promoter region and promotes CDX2 methylation by activating the WNT signaling pathway, leading to E-cadherin suppression in BC [40]. DGCR5 negatively regulates EMT by interacting with ARID1A, a chromatin-remodeling protein, to promote CDKN1A (P21) transcription and mediate cell proliferation and apoptosis. Thus, DGCR5 can potentially serve as a theranostic target for bladder cancer [69]. GATA6-AS epigenetically modulates endothelial gene expression by interacting with nuclear LOXL2 and impairing its function as an H3K4me3 deaminase, thus facilitating histone methylation and controlling endothelial cell functions [70]. Recently, genome-wide epigenetic reprogramming during EMT has been demonstrated [71]. Thus, additional studies identifying diverse lncRNA partners of epigenetic regulation will broaden our understanding of EMT and may lead to the development of targeted therapies.

#### 3.1.2. Transcriptional Activators

LncRNAs have been identified as a novel group of transcriptional activators that promote the transcription of EMT-related genes, which can affect the progression of various malignant tumors. Snail, one of the most significant EMT TFs, is frequently overexpressed in metastatic cancers. A novel lncRNA ELIT-1, Smad3, and Snail form a positive feedback loop by recruiting Smad3 (Sekelsky mothers against dpp 3) to the promoter of Snail, other TGF-β-target genes, and ELIT-1 itself, enhancing TGF-β/Smad3 signaling and promoting EMT progression in lung adenocarcinoma (LUAD) and GC [44]. In contrast, SATB2-AS1 suppresses colorectal cancer (CRC) progression by acting as a scaffold to recruit protein p300, whose acetylation of H3K27 and H3K9 at the SATB2 promoter to upregulate the expression of SATB2, a suppressor of CRC growth and metastasis. SATB2 subsequently recruits HDAC1 to the Snail promoter, inhibiting Snail transcription and repressing EMT in CRC [25]. LncRNA EPR enables epithelial cells to control proliferation by counteracting TGF-β-induced EMT. Mechanistically, EPR stimulates binding of SMAD3 and the mRNA decay-promoting factor KHSRP to the Cdkn1a promoter, which regulates Cdkn1a gene transcription and mRNA decay, respectively. Cdkn1a promotes cell cycle arrest in response to many stimuli, including TGF-β expression [45].

LncRNAs HOTTIP, BX111, and TBILA have been associated with EMT, mainly through their cross-talk with ZEB. HOTTIP directly binds adaptor protein WDR5 to the HOXA13 promoter locus and activates *HOXA13* gene transcription, resulting in upregulation of EMT-related TFs such as ZEB1. In addition, HOTTIP functions as a ceRNA to bind miR-30b, which promotes HOXA13 expression in esophageal squamous cell carcinoma (ESCC) [72] (Figure 3b). Additionally, BX111, induced by HIF-1α in response to hypoxia, is significantly increased in pancreatic cancer tissues and contributes to hypoxia-induced EMT. Mechanistically, BX111 activates ZEB1 transcription by recruiting the TF Y-box protein (YB1) to its promoter region, increasing ZEB1 expression and inhibiting downstream proteins E-cadherin and MMP2 [46] (Figure 3b). In non-small cell lung cancer, the TGFβ-induced lncRNA TBILA also promotes cell proliferation and metastasis by increasing ZEB1 expression [49].

In addition, lncRNA NBR2 is downregulated in osteosarcoma tissues and serves as a tumor suppressor gene by directly binding the Notch1 protein, thereby decreasing Notch1 mRNA expression and increasing E-cadherin mRNA expression [52]. Similarly, lncRNA NEF acts as an activator of its neighbor gene, FOXA2, which forms a positive-feedback loop in hepatocellular carcinoma (HCC). NEF, transcriptionally activated by FOXA2, physically interacts with β-catenin to increase GSK3β–β-catenin binding and thus promoting the inhibitory phosphorylation of β-catenin, resulting in FOXA2 upregulation and inhibited Wnt/β-catenin signaling, eventually suppressing EMT progression and cancer metastasis [53].

#### 3.1.3. Transcriptional Repressors

Recent findings indicate a role for lncRNAs as transcriptional repressors in a wide spectrum of malignant tumors. NEAT1, an estrogen-inducible lncRNA, is prerequisite for FOXN3 interactions with the SIN3A complex. FOXN3, as a transcriptional repressor, can bind the SIN3A repressor complex in estrogen receptor–positive cells. The FOXN3–NEAT1–SIN3A complex promotes EMT progression by transcriptionally repressing downstream target genes including GATA3 and TJP1. Interestingly, the FOXN3–NEAT1–SIN3A complex trans-represses the ER itself, forming a negative-feedback loop during transcriptional regulation. Increased expression of both FOXN3 and NEAT1 during BC progression corresponds to reduced GATA3 expression, and high levels of FOXN3 and NEAT1 are strongly associated with higher histological grades and poor prognosis [26] (Figure 3c). Similarly, HOTAIR and NEAT1 promote BC EMTs by regulating Twist and E-cadherin expression, respectively [26,73]. B3GALT5-AS1 directly binds to the miR-203 promoter, represses miR-203 transcription, upregulates miR-203 targets SNAI2 and ZEB2, and induces EMT progression in colon cancer (Figure 3c) [57]. In contrast, MALAT1 suppresses BC EMTs by acting as a scaffold to recruit ELAVL1 to the promoter region of CD133 (also known as PROM1) to downregulate CD133 expression. CD133, a marker of cancer stem cells, can promote EMT in various cancers [74]. This study indicates that the failure of a repressive complex to form or stabilize in breast cancer facilitates CD133 upregulation and an EMT-like program, providing new mechanistic insights into the control of pro-metastatic processes [75] (Figure 3c). Taken together, these findings indicate that lncRNAs regulate EMT progression by binding TFs or recruiting cofactors to the promoter regions of EMT-related genes. LncRNAs also directly bind the promoters of EMT-related genes, further promoting or inhibiting transcription to manipulate EMT states.

### 3.2. LncRNAs in EMTs at the Post-Transcriptional Level

#### 3.2.1. Interactions with miRNAs

MiRNAs play promoting and suppressive roles in a plethora of biological processes [76]. Recent studies have shown that lncRNAs act as ceRNAs to bind miRNAs and facilitate EMT-related gene expression. Here, we summarize recent advances in understanding lncRNAs as ceRNAs, based on the classification of EMT markers.

SNAI1 and SNAI2 (also known as Slug), two members of the Snail superfamily of zinc-finger transcriptional repressors, participate in EMT development and other processes [77]. Snail is most widely accepted as a suppressor of E-cadherin expression, and an increasing number of lncRNAs are being reported to regulate its expression. As mentioned above, lncRNA CAR10 and HCP5 induce LUAD EMT by directly binding miR-203 and regulating both SNAI1 and SNAI2 [59,78] (Figure 3d). Similarly, the oncogenic lncRNA TTN-AS1 facilitates expression of Snail1 and the actin-binding protein Fascin homolog 1 (FSCN1) by competitively binding miR-133b, resulting in ESCC cell proliferation, EMT, and metastasis [79]. TINCR and UCA1 also significantly upregulate Snail1 and Slug, respectively, and promote BC EMT [58,80]. Furthermore, TINCR is a potential prognostic indicator and therapeutic target molecule because it can promote trastuzumab resistance by binding miR-125b [80].

ZEB1 and ZEB2 have emerged as key regulators of E-cadherin and are associated with the malignancy of various human tumors, including CRC, HCC, and BC [27,61,81]. LncRNA PNUTS acts as a ceRNA to competitively bind miR-205, which targets ZEB during EMT. Importantly, PNUTS production is regulated by hnRNP E1 binding to an alternative splicing site in the PNUTS pre-RNA [81]. Through alternative splicing, the PNUTS gene can encode either an mRNA or lncRNA, the latter of which competitively binds miR-205 to promote EMT [81]. Similarly, lncRNA AK000053 competitively interacts with miR-508 and promotes the expression of miR-508-targeted genes (Zeb1, Bmi1, and Sall4), resulting in altered EMT properties [61]. 

Twist is a basic helix-loop-helix (bHLH) protein that is transcriptionally active during lineage determination and cell differentiation [82]. It exerts a well-established role in inducing EMT to facilitate tumor invasion and metastasis [83]. Lnc-ATB was first reported to be abundant in HCC cells after stimulation with TGF-β and a prognostic marker of HCC patient survival [27]. Strikingly, since its discovery in HCC, lnc-ATB has been found to competitively bind miR-200s to restore ZEB1 and ZEB2 expression or promote IL-11 signaling [27]. In BC, lnc-ATB facilitates EMT by binding miR-200c to increase Twist1 expression [84].

Previous studies have also indicated that lncRNAs regulate EMT by indirectly increasing the expression of Snail, ZEB, or Twist. E-cadherin, occludins, and cytokeratins are the most common epithelial markers, and N-cadherin and vimentin are the most common mesenchymal markers [85]. Some experimental models require a dramatic change in the expression of select epithelial and mesenchymal markers to distinguish between EMT states [86]. SNHG3 and LUCAT1 are increased in HCC promote EMT by competitively binding miR-128 and miR-301b, respectively, to regulate E-cadherin and N-cadherin expression [87,88]. Importantly, SNHG3 is a novel biomarker and therapeutic target for forecasting sorafenib responses by regulating drug resistance [87]. In addition, LUCAT1 can form a positive feedback loop with STAT3 and miR-301b to promote cell proliferation, migration, and invasion. STAT3 binds to the LUCAT1 promoter region and enhances its transcription. LUCAT1 increases STAT3 expression by competitively binding miR-301b [88]. Recent study suggested that ceRNAs are inherent regulatory components of EMT and represent potential targets for interfering with EMT during tumorigenesis [89]. Taken together, these findings show that lncRNAs can affect EMT and mediate drug resistance as ceRNAs. Hence, it is crucial to extend the analyses of ceRNAs to the dynamic biological processes in EMT to further elucidate the role of ceRNA-based regulation and obtain novel insights into possible tumor therapies.

Exosomes are a subtype of extracellular vesicles that are frequently associated with tumor progression [90]. Emerging evidence suggests that exosomal lncRNAs play significant roles in regulating tumor progression, including EMT. For example, the exosomal lncRNA Sox2ot is a ceRNA that can promote pancreatic ductal adenocarcinoma (PDAC) EMT and induce stem cell-like properties by competitively binding miR-200s to increase expression of the neighboring Sox2 gene. Moreover, high plasma exosomal Sox2ot expression correlates with the tumor node metastasis (TNM) stage and overall survival rate of patients with PDAC [91] (Figure 3d). Besides ceRNA, exosomal lnc-MMP2-2 can promote EMT by acting as an “enhancer- like lncRNA” and can bind to the upstream site of MMP-2 gene, resulting in augmentation of MMP2 expression in TGF-β- mediated lung cancer invasion and also increasing vascular permeability [92]. In addition, hypoxia enhances exosome-mediated reciprocal movement of the lncRNA UCA1 into bladder cancer cells, which facilitates cancer growth and progression by inducing EMT [93]. Similarly, the exosomal lncRNA ZFAS1 enhances GC cell migration and EMT involved in lymphatic metastasis [94]. In contrast, NONHSAT105177 trafficking is mediated by exosomes and inhibits PDAC cell proliferation, migration, and EMT [95]. Taken together, these findings suggest that gaining mechanistic insight into how exosomal lncRNAs can regulate EMT and may lead to treatments for various diseases. Therefore, circulating exosomal lncRNAs may serve as liquid biopsy and non-invasive biomarkers for the early detection, diagnosis, and treatment of diseases.

#### 3.2.2. Regulation of mRNA Stability and Splicing

Recently, lncRNAs were reported to participate in mRNA regulation by affecting mRNA translation, storage, and degradation. Interestingly, lncRNAs directly regulate EMT markers by increasing their stabilities. LncRNA CASC11, an oncogene, facilitates osteosarcoma (OS) metastasis and EMT by associating with Snail mRNA and inhibiting Snail degradation. CASC11 binding to the Snail mRNA 3′- untranslated region (UTR) blocks the suppression of miR-122, miR-145, miR-211, miR-34a, and miR-137 [96] (Figure 3e). LncRNA TTN-AS1 regulates mRNA stability by interacting with HuR, which is an omnipresent RNA binding protein (RBP) associated with specific mRNAs that promotes their stability [97,98]. Mechanistically, HuR directly interacts with both TTN-AS1 and FSCN1 mRNA, resulting in upregulation of FSCN1 and β-catenin protein, thereby correlating with ESCC invasion and EMT progression [79]. Similarly, the lncRNA AC132217.4 was remarkably up-regulated and promoted oral squamous cell carcinoma (OSCC) migration and EMT. Mechanistically, KLF8 increases AC132217.4 transcription by binding to its promoter. LncRNA AC132217.4 interacts with the 3′-UTR of IGF2 mRNA and activates AKT signaling by increasing IGF2 mRNA stability [43] (Figure 3e). Furthermore, lnc-ATB facilitates the HCC invasion-metastasis cascade by binding IL-11 mRNA, increasing its stability, causing autocrine induction of IL-11, and activating STAT3 signaling [27]. Recent evidence shows that lncRNA ZEB2-AS1 (also known as ZEB2AS, ZEB2-AS, ZEB2NAT) modulates the invasion and EMT progression in bladder cancer as well as in gastric cancer [99,100]. Strikingly, ZEB2-AS1 (Zeb2-NAT) can also regulate the mRNA splicing to impair EMT. Mechanistically, ZEB2-AS1 prevents splicing of the Zeb2 5′-UTR in epithelial cells, increases the levels of Zeb2 protein, and consequently down-regulates E-cadherin mRNA and protein [101] (Figure 3e). With increasing recognition that lncRNAs play positive and negative regulatory roles in disease processes [102], pharmacological efforts in targeting lncRNAs might result in the development of novel anti-tumor therapeutics.

#### 3.2.3. Protein and mRNA Modifications

##### 3.2.3.1. Protein Modification

LncRNAs are also diverse regulators of protein modification processes, controlling gene expression and function at the post-transcriptional level. Protein modifications, including phosphorylation and ubiquitination, have been used to identify post-translational modifiers that regulate many aspects of EMT. Phosphorylation is one of the most important post-translational protein modifications; it is related to the regulation of many vital activities. As a reaction catalyzed by protein kinases, it functions significantly in cell signaling. LncRNA MUF, which is highly expressed in HCC, activates Wnt/β-catenin signaling and induces EMT by binding annexin A2 and altering the subcellular localization of β-catenin, resulting in β-catenin phosphorylation and Wnt cascade activation [47]. In CRC, SLCO4A1-AS1 activates Wnt/β-catenin signaling through enhancement of the stability of β-catenin, by attenuating the interaction of β-catenin with GSKβ and inhibiting β-catenin degradation [54]. Similarly, the heterotrimeric complex composed of CYTOR, NCL, and Sam68 activates the NF-κB pathway and promotes EMT, thereby contributing to CRC progression. CYTOR mediates the interaction of NCL and Sam68 in its EXON1, which increases phosphorylation of P65 [51] (Figure 3f). These findings provide strong clinical evidence to support using CYTOR as a biomarker of CRC recurrence and prognosis. Additionally, based on the importance of the NCL-CYTOR-Sam68 complex, these molecules might serve as novel targets for CRC therapies.

Ubiquitination, a versatile molecular signature, has been found to dynamically modulate protein functions. LncRNA SNHG15 transcription is upregulated, and ectopic expression of SNHG15 promotes colon cancer cell migration, accelerating xenografted tumor growth. SNHG15 interacts with Slug and blocks Slug degradation via the ubiquitin–proteasome pathway [48] (Figure 3f). Another example, lncRNA GAEA associates with the RNA binding E3 ligase MEX3C and enhances its enzymatic activity, resulting in K27-linked polyubiquitination (PolyUb) of PTEN. The PTENK27-PolyUb complex removes phospho groups from serine/threonine residues in various substrates, including TWIST1, SNAI1, and YAP1. Upon dephosphorylation, these proteins are stabilized and promote EMT in a PTEN-dependent manner [29]. Similarly, LINC01638 is highly expressed in triple-negative breast cancer (TNBC) tissues and cells. LINC01638 interacts with c-Myc to block SPOP-induced c-Myc ubiquitination and degradation, and then stimulates MTDH-Twist1 signaling to maintain mesenchymal characteristics with EMT and CSC-like features [50]. 

LncRNAs have also been identified as a group of regulators of EMT by regulating other protein modifications, such as competitive protein-binding sites and the inhibition of nuclear entry. In CRC, LINC01133 and CRCMSL function as EMT-suppressor genes by competing with SRSF6 protein binding sites and inhibiting nuclear translocation of high mobility-group box 2 (HMGB2), respectively. LINC01133 is downregulated by TGF-β and suppresses EMT by binding to SRSF6 and blocking the function of its critical domain [55]. lnc-CRCMSL acts as an anti-metastasis gene that suppresses EMT by physically binding to HMGB2 and stabilizing the retention of HMGB2 in the cytoplasm to weaken the HMGB2-OCT4 interactions [56]. Together, while functional lncRNA research has illustrated important associated post-translational modifications, many intriguing issues remain concerning how lncRNAs obstruct the modifications of diverse biological proteins. 

##### 3.2.3.2. mRNA Modification

The mRNA modification has opened a new realm of post-transcriptional gene regulation, of which most abundant internal mRNA modification is m^6^A [103,104,105]. A recent study showed a new link between m^6^A and lncRNAs that modulates all phases of the RNA life cycle associated with expression. LncRNA FOXM1-AS can promote the interaction of ALKBH5 with FOXM1 nascent transcripts to maintain tumorigenesis [106]. Studies on the biological functions of m^6^A modification during EMT progression have also been reported. As described above, m^6^A can regulate EMT progression by triggering the translation of Snail, via YTHDF1 and eEF-2 binding with methylated mRNA [107]. Modification with m^6^A in the Snail coding sequence (CDS) but not the 3′-UTR induces polysome-mediated translation of Snail mRNA in liver cancer. Research has also shown that m^6^A-modified lncRNA RP11 positively regulates CRC migration and EMT and enhances liver metastasis by upregulating Zeb1. Post-translational upregulation of Zeb1 is essential for RP11-induced CRC cell dissemination. Mechanistically, m^6^A causes RP11 to accumulate in the nucleus and on chromatin. RP11 stimulates Zeb1 expression by downregulating Siah1 and Fbxo45 mRNAs by binding to hnRNP A2B1 [28] (Figure 3f). This finding suggests that RP11 could be used as a predictive biomarker of CRC metastasis and an effective target for anti-metastatic therapies. Because our understanding of the mechanisms underlying RNA methylation is still in its infancy, additional research of regulatory patterns mediated by m^6^A modification of lncRNA is warranted.

## 4. EMT Pathways Control by lncRNAs

EMT can be induced by the activation of various signaling pathways when epithelial cells encounter specific signals released by the cells forming their stromal microenvironment [86,108]. The binding of ligands of stromal origin to their cognate receptors expressed by normal and neoplastic epithelial cells triggers consecutive signal-transduction pathways that ultimately converge and activate the EMT program. The cellular adaptations characterizing the EMT hallmarks can be driven by growth factor signaling pathways, such as transforming growth factor β (TGF-β), Wnt (Wingless/Integrated), fibroblast growth factor (FGF), and Notch. These pathways instruct the expression and activity of EMT-TFs, which operate in coordination with changes of gene expression in order to modulate the EMT [109].

**Figure 4:**
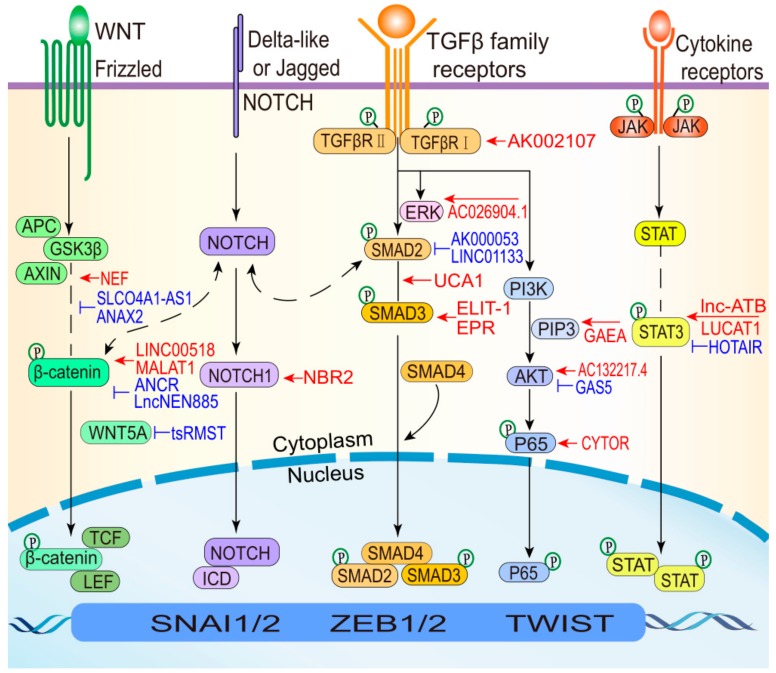
LncRNAs regulate EMT signaling pathways. When the canonical Wnt pathway is activated, β-catenin is released from the GSK3β-AXIN-APC complex. Then, β-catenin translocates to the nucleus and drives EMT. Thus, lncRNAs regulate this pathway by targeting β-catenin, resulting in EMT induction (red) or inhibition (blue). Additionally, lncRNAs can modulate non-canonical Wnt signaling to suppress EMT by repressing WNT5A (e.g., tsRMST). The Notch pathway controls cell fate decisions, differentiation, and proliferation. LncRNAs can inhibit EMT by regulating Notch1 signaling (e.g., NBR2). In TGF-β pathway, TGF-β-induced SMAD (Sekelsky mothers against dpp) complexes transcriptionally activate EMT TFs. Once they are activated, EMT TFs can increase the expression of TGF-β ligands and drive a positive feedback loop, thereby helping cells to maintain an EMT state. Thus, lncRNAs can regulate EMT through SMAD2 (e.g., AK000053 and LINC01133), SMAD3 (e.g., ELIT-1 and EPR), and the SMAD2/3 complex (e.g., UCA1), and TGFBR1 (e.g., AK002107). Alternate pathways involve collaboration between TGF-β and proteins such as ERK, PI3K-AKT, and NF-κB (P65), which are also regulated by lncRNAs (e.g., AC026904.1, GAEA, AC132217.4, and CYTOR). STAT3 in the STAT pathway is a key TF that determines the EMT state and tumor aggression. LncRNAs can impact EMT via STAT3 activation (red) or inactivation (blue).

During EMT, lncRNAs induce the expression of various EMT TFs and alter critical molecules to regulate wound healing, development and tumor metastasis involving pathways, including the TGF-β, Wnt, and STAT3 pathways (Figure 4). The TGF-β pathway plays a central role in inducing EMT and can collaborate with several other signaling pathways (e.g., ERK, PI3K-AKT, and NF-κB) contributed to EMT programs. For example, lncRNA UCA1 and AC026904.1 are upregulated through the TGF-β/Smad and TGF-β/ERK pathways, respectively, to induce EMT in BC [58]. The Wnt signaling pathway is one of the most important pathways that is hyperactivated in cancers [110]. Furthermore, the Wnt pathway plays important roles in inducing different EMT-related phenotypes, culminating mainly in the nuclear translocation of β-catenin. LncRNA NEF and ANCR suppress Wnt/β-catenin signaling by manipulating the key protein β-catenin, which inhibits EMT [41,53]. STAT3 activation is crucial for its mediation of cytokine and growth factor-induced cellular and biological processes involved in cell development, proliferation, survival, and inflammation [111,112]. Aberrations in the STAT3 pathway are intimately associated with the development of diverse cancer types [113,114]. For instance, lnc-ATB activates STAT3 signaling to promote HCC EMT by increasing IL-11 mRNA stability [27]. In addition to the aforementioned well-known pathways, other signaling pathways can participate in the induction of different EMT states in various cellular contexts. Recent studies have revealed that the lncRNA-associated NF-κB and Notch pathways regulate EMT states. Previous reports have also shown that certain drugs can inhibit cancer metastasis by blocking EMT in malignant cells and reduce drug susceptibility by deactivating EMT-related pathways, including the AKT and STAT3 signaling pathways [115,116,117]. Therefore, developing inhibitors that target lncRNAs could effectively control EMT-related processes. Interrupting interactions between lncRNAs that have specific expression characteristics in different EMT states and various signaling pathways represent a novel means of controlling the EMT process. 

## 5. LncRNAs as Biomarkers and Therapeutic Targets

LncRNAs are uniquely expressed in differentiated tissues or tumor types in a spatiotemporal manner. Thus, many lncRNAs are useful for understanding the molecular mechanisms underlying physiological and pathological processes. Indeed, some lncRNAs are dysregulated in tumor samples compared with normal tissues and have remarkable correlations with EMT, and migration, such as H19 in esophageal cancer and breast cancer [118,119]. Accumulating reports indicated that the distribution and levels of lncRNAs are condition-specific in diverse locations, which have been exploited as promising prognostic and diagnostic biomarkers for cancers [120]. Currently, lncRNA PCA3 has been approved by FDA (Food and Drug Administration) as an early diagnostic biomarker of prostate cancer [121]. Furthermore, emerging studies have shown that signatures composed of combinations of various circulating lncRNAs can increase the efficiency of cancer detection [122]. In preclinical cancer models, antisense oligonucleotides (ASOs) have already proved to be useful in modulating gene expression linked to various diseases, such as lymphoma, and solid tumors [123]. Preclinical models with lncRNA MIR31HG outlier expression were characterized by reduced expression of MYC targets as well as elevated EMT, TNF-α/NFκB, TGF-β, and IFN-α/γ gene expression signatures, thereby indicating cancer cell-intrinsic properties resembling the CMS4 subgroup-associations which were recapitulated in patient biopsies [124]. Additionally, exosomal lncRNAs that promote EMT and accelerate the proliferation of cancer cells are associated with the occurrence and progression of cancer and can be used for diagnosis and treatment [91,93,125]. Numerous cancers frequently become resistant to chemotherapeutic agents. In these chemotherapy-resistant tumors, dysregulated lncRNAs, including lncRNA SNHG3, and TINCR [80,87], contribute noticeably to the development of drug resistance. Understanding the molecular mechanisms through which lncRNAs function is crucial for cancer diagnosis and prognosis, developing novel therapeutics, and predicting responses to treatment with EMT modulators.

## 6. Conclusions and Perspectives

LncRNAs directly and indirectly modulate EMT progression by targeting diverse EMT markers, which play a crucial role in all types and states of EMTs, therefore contributing to tumorigenesis and tumor progression, as well as drug resistance [85,126,127]. The mechanisms whereby lncRNAs regulate EMT can be summarized as follows: (i) In the epigenetic layer, lncRNAs recruit epigenetic factors to orchestrate the expression of EMT-related genes. (ii) At the transcriptional level, lncRNAs regulate the expression of EMT-related genes by binding TFs and promoters. (iii) At the post-transcriptional level, EMT markers are regulated by lncRNA competitively binding miRNAs, regulating mRNA stability and splicing, and modifying RNA or proteins. Furthermore, EMT-related signaling pathways are strengthened or weakened by interactions with lncRNAs and cascade molecules. EMT regulation by lncRNAs, therefore, is an extensive and complex process that occurs during the progression of several human diseases.

LncRNA expression levels are stringently restricted spatiotemporally in diverse and heterogeneous tissues. The high tissue specificity enables fine-scale gene regulation and likely underpins condition-specific differences in function. Indeed, many lncRNAs have been depicted as having different and often conflicting functions in different types of EMT and cancer regulation, such as the lncRNA XIST, ZFAS1, and OIP5-AS1 [128,129,130,131,132,133]. Recent findings have indicated that lncRNAs exert dual functions involving promotion and inhibition of controlling EMT. LncRNA XIST and ZFAS1 promote CRC cell proliferation, invasion, and EMT by competitively binding miRNAs to upregulate ZEB1 expression. In contrast, other studies indicate that XIST and ZFAS1 induce protective factors to inhibit EMT progression in BC [128,129,130,131]. Similarly, lncRNA OIP5-AS1 facilitates EMT by increasing ZEB1 translation but inhibits EMT in diverse range of cancers [132,133]. Together, the dual effects of lncRNAs on EMT are associated with their expression levels, indicating that lncRNAs may serve as complex prognostic factors in various human diseases.

Numerous studies have indicated that EMT progression contributes to early-stage dissemination and is key for cancer invasion and metastasis [9,134]. However, some studies have shown that EMT is not required for metastasis in certain tumors [135,136]. A confusing study revealed that lncRNA B3GALT5-AS1 induced EMT but suppressed colon cancer liver metastasis [57]. In liver metastatic foci, metastasized colon cancer cells undergo a mesenchymal-endothelial transition (MET), the reverse of EMT, and regain the epithelial phenotype to permit their settlement and proliferation at distant sites [137]. Recent studies have identified four characteristics that are required for metastasis: cell motility and invasion potential, the ability for secondary sites or local microenvironments to be modulated, cell plasticity, and the ability of cancer cells to colonize secondary tissues. EMT is not essential for the completion of the metastatic cascade in every tumor type [138]. Currently, EMT is viewed as a focal rather than a global event, and probably a response of tumor cells to their local microenvironment [8]. Moreover, the opposing roles of EMT in the early invasion and late settlement stages of colon cancer metastasis to the liver imply that disease stage-specific therapies are essential. The latest research broadens our understanding of EMT in metastasis and demonstrates that heterogeneous EMT phenotypes are important parameters for tumor prognosis and treatment [139]. Thus, EMT features can preclinically suggest combined therapies that may eradicate transformed cell populations at different stages.

A better understanding of how lncRNAs regulate EMT progression at different molecular levels can drive innovative anti-metastasis therapeutic strategies and also identify prognostic or diagnostic markers for a diverse range of diseases. Additionally, attaining a greater understanding of how various EMT states can be regulated by lncRNAs and how their complex control mechanisms are interconnected with different signal transduction pathways may also suggest ways to limit tumor progression. We believe that targeting lncRNAs will lead to novel effective drugs and other therapeutic approaches to control different EMT states in diverse diseases.

## Figures and Tables

**Table 1:** Examples of lncRNAs that interact with various molecules in Epithelial-mesenchymal transition (EMT).

Category	LncRNA	Partners	Expression	EMT Markers	Pathways	Function	Mechanism	Tumor Types	Reference
lncRNA: chromatin regulators	MEG8	EZH2	↑	SNAI1/2	TGF-β	Promote EMT	Interacts with EZH2 and represses miR-34a and miR-203, resulting in up-regulated of SNAI1/2.	Lung and pancreatic cancer	[15]
	MEG3	EZH2	↑	ZEB family	TGF-β	Promote EMT	Interacts with JARID2 and EZH2 and represses CDH1 and miRNA-200 family, resulting in up-regulated of ZEB.	Lung cancer	[39]
	LINC00518	CDX2	↑	ZEB1/2 Twist1	Wnt	Inhibit EMT	Binds to the promoter region of CDX2 gene and promotes CDX2 methylation by recruiting DNA methyltransferase through activating the Wnt signaling pathway.	Breast cancer	[40]
	MALAT1	Ezh2	↑	E-cadherin	Wnt/β-catenin	Promote EMT and metastasis.	Activated by c-Fos and interacts with Ezh2, resulting in E-cadherin expression was decreased.	RCC	[13]
	ANCR	EZH2	↓	E-cadherin	Wnt/β-catenin	Inhibit EMT and metastasis.	Interacts with EZH2 to increase the binding of CDK1 with EZH2 and to promote the degradation of EZH2, resulting in the up-regulated of E-cadherin.	Breast cancer	[41]
lncRNA promoter: TFs	MALAT1	STAT3	↑	Snail	TGF-β/STAT3	Promote EMT	STAT3 binds to the MALAT1 promoter region and transcriptionally activate MALAT1 expression.	HNSCC	[14]
	HCCL5	ZEB1	↑	ZEB1 E-cadherin	TGF-β1	Promote EMT and metastasis.	ZEB1 can bind to both the identified super-enhancer and promoter of HCCL5. HCCL5 was significantly and frequently overexpressed.	HCC	[42]
	AC132217.4	KLF8	↑	E-cadherin	AKT	Promote EMT	KLF8 binds to the upstream sequence of AC132217.4, activating its expression at the transcriptional level.	OSCC	[43]
lncRNA: TFs	ELIT-1	Smad3	↑	Snail	TGF-β	Promote EMT	Binds to Smad3 and enhances Smad -responsive promoter activities by recruiting Smad3 to the promoters of its target genes including Snail, other TGF-β-target genes, and ELIT-1 itself.	lung adenocarcinoma gastric cancer	[44]
	EPR	SMAD3	↓	SNAI1	TGF-β	Inhibit EMT	Interacts with SMAD3 and promotes Cdkn1a gene expression, resulting in the down-regulation of SNAI1.	Breast cancer	[45]
	tsRMST	NANOG, SUZ12	↓	SNAI2 TWIST1	Wnt	Inhibit EMT	Binds to NANOG and SUZ12 to repress the expression of WNT5A, resulting in the down-regulation of SNAI2 and TWIST1.	hESCs	[31]
	BX111	YB1	↑	ZEB1 MMP2 E-cadherin	—	Promote EMT and metastasis.	Activates transcription of ZEB1 via recruiting YB1 to its promoter region, resulting in the up-regulation of ZEB1.	Pancreatic cancer	[46]
	NEAT1	FOXN3	↓	E-cadherin	—	Inhibit EMT	Interacts with FOXN3 and SIN3A and represses the target genes including GATA3 and TJP1, resulting in the up-regulated of E-cadherin.	Breast cancer	[26]
lncRNA:Protein	MUF	ANXA2	↑	Snail	Wnt	Promote EMT	Binds to the protein ANXA2 and ANXA2 can alter the subcellular localization of β-catenin to activate the Wnt cascade.	HCC	[47]
	SNHG15	Slug	↑	Slug	—	Promote EMT	Interacts with protein Slug via its C-terminal domain containing five zinc finger motifs, and promote Slug expression.	colon cancer	[48]
	TBILA	S100A7	↑	SNAI1 ZEB1,	S100A7/JAB1	Promote EMT	Binds to the S100A7 protein and promotes S100A7/JAB1 pathway activation, resulting in the up-regulation of SNAI1 and ZEB1.	NSCLC	[49]
	RP11	hnRNPA2B1	↑	Zeb1	—	Promote EMT	Interacts with the protein hnRNPA2B1 and accelerates the mRNA degradation of Siah1 and Fbxo45, and subsequently prevented the proteasomal degradation of Zeb1.	CRC	[28]
	GAEA	MEX3C	↑	SNAI1 TWIST1,	AKT	Promote EMT	Binds to the MEX3C and catalyze K27-linked polyUb of PTEN. PTEN^K27-PolyUb^ removed phospho-groups from serine/threonine residues in substrates including TWIST1, SNAI1, and YAP1.	Human and mouse breast epithelial cells	[29]
	LINC01638	c-Myc	↑	Twist1	MTDH-Twist1	Promote EMT	Interacts with protein c-Myc to prevent SPOP-induced c-Myc ubiquitination and degradation and then activate MTDH-Twist1 signaling to maintain mesenchymal traits with EMT and CSC-like features.	TNBC	[50]
	CYTOR	NCL Sam68	↑	Twist E-cadherin	NF-ΚB	Promote EMT	NCL and Sam68 could recognize their specific motifs and directly bind to Exon1 of CYTOR, then activating the NF-κB pathway and promoting the expression of Twist.	CRC	[51]
	NBR2	Notch1	↓	E-cadherin	Notch	Inhibit EMT	Binds to the Notch1 protein and promotes Notch1 expression, resulting in the up-regulation of E-cadherin.	osteosarcoma	[52]
	NEF	β-catenin	↓	E-cadherin	Wnt	Inhibit EMT and metastasis.	Activated by FOXA2 and can interact with β-catenin, leading to the suppression on Wnt/β-catenin signaling and activation of FOXA2 expression.	HCC	[53]
	SLCO4A1-AS1	β-catenin	↑	E-cadherin	Wnt	Promote EMT and metastasis.	Activates Wnt signaling through enhancing the stability of β-catenin by attenuating the interaction of β-catenin with GSKβ.	CRC	[54]
	LINC01133	SRSF6	↓	E-cadherin	TGF-β	Inhibit EMT and metastasis.	Binds to SRSF6 and blocking its critical domain, resulting in inhibition of EMT.	CRC	[55]
	CRCMSL	HMGB2	↓	OCT4	—	Inhibit EMT and metastasis.	Binds to protein HMGB2 and stabilizes the localization in the cytoplasm, attenuating the interaction between HMGB2 and OCT4 and inhibiting EMT.	CRC	[56]
lncRNA: miRNA	B3GALT5-AS1	miR-203	↓	ZEB2, SNAI2	—	Promote EMT and metastasis.	Directly binds to the promoter of miRNA-203 and represses miR-203 expression, resulting in the up-regulated of ZEB2 and SNAI2.	Colon cancer	[57]
	UCA1	miR-1, miR-203a	↑	Slug	TGF-β	Promote EMT	Promotes Slug expression at the post- transcriptional level, by directly titrating miR-1 and miR-203a.	Breast cancer	[58]
	CAR10	miR-30, miR-203	↑	SNAI1/2	—	Promote EMT and metastasis.	Induces EMT by directly binding with miR-30 and miR-203 and then regulating the expression of Snail1 and Slug.	LUAD	[59]
	FTX	miR-374a	↑	Snail ZEB1	Wnt/β-catenin	Promote EMT	Competitively binding miR-374a, thus resulting in the up-regulated of Snail and ZEB1	HCC	[60]
	lnc-ATB	miR-200s	↑	ZEB1/2	TGF-β	Promote EMT	lnc-ATB upregulated ZEB1 and ZEB2 by competitively binding the miR-200 family and then induced EMT and invasion.	HCC	[27]
	AK000053	miR-508	↑	ZEB1	TGF-β	Promote EMT	Competitively interact with miR-508 and negatively regulated, resulting in the up-regulated of ZEB1.	CRC	[61]
	ZFAS1	miR-150	↑	ZEB1, MMP14/16	—	Promote EMT and metastasis.	Competitively binding miR-150, resulting in the up-regulated of ZEB1, MMP14/16	HCC	[62]
lncRNA: mRNA	RP11	Fbxo45, Siah1	↑	Zeb1	—	Promote EMT	Interacted with the 3’UTR of Fbxo45 mRNA and CDS of Siah1 mRNA, and subsequently prevented the proteasomal degradation of Zeb1.	CRC	[28]
	AC132217.4	IGF2	↑	E-cadherin	AKT	Promote EMT and metastasis.	Interacted with the 3’UTR of IGF2 mRNA and activated AKT signalling by increasing IGF2 mRNA stability, remarkably down-regulated of E-cadherin.	OSCC	[43]
	lnc-ATB	IL-11	↑	E-cadherin	IL-11/STAT3	Promote EMT	Binds to IL-11 mRNA, thus increasing IL-11 mRNA stability, causing autocrine induction of IL-11, and then activating STAT3 signaling.	HCC	[27]

RCC: renal cell carcinoma; BC: breast cancer; HNSCC: head and neck squamous cell carcinoma; HCC: hepatocellular carcinoma; OSCC: oral squamous cell carcinoma; hESCs: human pluripotent stem cells; ESCC: esophageal squamous cell carcinoma; NSCLC: non-small cell lung carcinomas; TNBC: triple-negative breast cancer; LUAD: Lung adenocarcinoma; PADC: pancreatic ductal adenocarcinoma.
